# Respiratory bioenergetics is enhanced in human, but not bovine macrophages after exposure to *M. bovis* PPD: Exploratory insights into overall similar Cellular Metabolic Profiles

**DOI:** 10.1177/17534259241296630

**Published:** 2024-11-20

**Authors:** Marie-Christine Bartens, Sam Willcocks, Dirk Werling, Amanda J Gibson

**Affiliations:** 1 Centre for Vaccinology and Regenerative Medicine, Department of Pathobiology and Population Science, Royal Veterinary College, Hatfield, UK; 2 Department of Infection Biology, London School of Hygiene and Tropical Medicine, UK; 3 Department of Life Sciences, Brunel University, UK; 4 Department of Life Science, Aberystwyth University, UK

**Keywords:** Macrophage, immunometabolism, tuberculosis, mycobacteria, BCG

## Abstract

The role of macrophage (MØ) cellular metabolism and reprogramming during TB infection is of great interest due to the influence of *Mycobacterium* spp. on MØ bioenergetics. Recent studies have shown that *M. tuberculosis* induces a TLR2-dependent shift towards aerobic glycolysis, comparable to the established LPS induced pro-inflammatory M1 MØ polarisation. Distinct differences in the metabolic profile of murine and human MØ indicates species-specific differences in bioenergetics. So far, studies examining the metabolic potential of bovine MØ are lacking, thus the basic bioenergetics of bovine and human MØ were explored in response to a variety of innate immune stimuli. Cellular energy metabolism kinetics were measured concurrently for both species on a Seahorse XFe96 platform to generate bioenergetic profiles for the response to the bona-fide TLR2 and TLR4 ligands, FSL-1 and LPS respectively. Despite previous reports of species-specific differences in TLR signalling and cytokine production between human and bovine MØ, we observed similar respiratory profiles for both species. Basal respiration remained constant between stimulated MØ and controls, whereas addition of TLR ligands induced increased glycolysis, as measured by the surrogate parameter ECAR. In contrast to MØ stimulation with *M. tuberculosis* PPD, another TLR2 ligand, *M. bovis* PPD treatment significantly enhanced basal respiration rates and glycolysis only in human MØ. Respiratory profiling further revealed significant elevation of ATP-linked OCR and maximal respiration suggesting a strong OXPHOS activation upon *M. bovis* PPD stimulation in human MØ. Our results provide an exploratory set of data elucidating the basic respiratory profile of bovine vs. human MØ that will not only lay the foundation for future studies to investigate host-tropism of the *M. tuberculosis* complex but may explain inflammatory differences observed for other zoonotic diseases.

## Highlights

- Similar baseline respiratory profiles for human and bovine macrophages
- *M. bovis* PPD treatment altered metabolic profile only in human MØ
- Strong OXPHOS activation upon *M. bovis* PPD stimulation only in human MØ

## Introduction

In the recent years, a growing interest in the cellular metabolism of innate immune cells has developed due to the understanding that changes in metabolic pathways of macrophages (MØ) in response to agonist stimulation impact on their phenotype and function.^1–3^ Numerous studies have emphasised that glycolysis, and therefore the provision of energy is crucial for immune cell function.^2^ Indeed, stimulation of MØ with various pattern recognition receptor (PRR) ligands, most commonly lipopolysaccharide (LPS), induces a metabolic shift from oxidative phosphorylation (OXPHOS) to glycolysis. This is considered a hallmark event in MØ activation, similar to the Warburg effect known in tumour cells.^1,4^

The Warburg effect occurs in tumour cells under normoxic conditions and glycolysis is the dominant metabolic pathway.^5^ During glycolysis, glucose is converted to pyruvate that enters the tricarboxylic acid cycle (TCA) cycle before being subsequently further metabolised by OXPHOS in the mitochondria. In tumour cells, pyruvate is metabolised to lactate instead of entering the TCA. Similar to this effect, activation of MØ induces a similar metabolic shift, with increased glycolysis, reduction in TCA cycle activity^6,7^ and increased lactate production and flux through the pentose phosphate pathway (PPP) (reviewed by Kelly and O’Neill^4^).

OXPHOS, like glycolysis, results in ATP production, though in significantly lesser magnitude. However, glycolysis can generate ATP more rapidly, which is important for MØ effector functions under acute stress such as during pathogen infection, particularly host defence functions including phagocytosis and production of inflammatory cytokines.^2^

Altered MØ metabolism upon LPS plus interferon-γ (IFN-γ) stimulation, in comparison to interleukin-4 (IL-4) stimulation, forms the basis of MØ priming into either pro-inflammatory, classically activated M1 MØ or anti-inflammatory, alternately activated M2 MØ.^1,2^ This metabolic programming leads to M1 MØ being associated with host defence pathways, whereas M2 MØ promote T-helper cell type 2 (T_H_2) driven immune responses and modulate repair processes.^3^ While this M1 – M2 dichotomy may be somewhat extreme, there are ligands such as LPS and the synthetic diacylated lipoprotein FSL-1, a TLR2/TLR6 ligand that can be used to induce a more M1 or more M2 biased phenotype.^8^ The main metabolic characteristics of M1 MØ are strongly enhanced glycolysis and impaired OXPHOS, similar to the Warburg effect described above. In combination with an enhanced PPP metabolism, this supports the resourcing of nucleotides for protein synthesis and increased nicotinamide adenine dinucleotide phosphate hydrogen (NADPH) production for inflammatory MØ responses. Subsequent oxidation of NADPH results in the production, and release, of reactive oxygen species (ROS), facilitating a direct bactericidal effect of MØ.^9^ To prevent hyper-inflammation of the tissue, NADPH is also used to generate glutathione and other antioxidants.^2^

**Figure 1.**
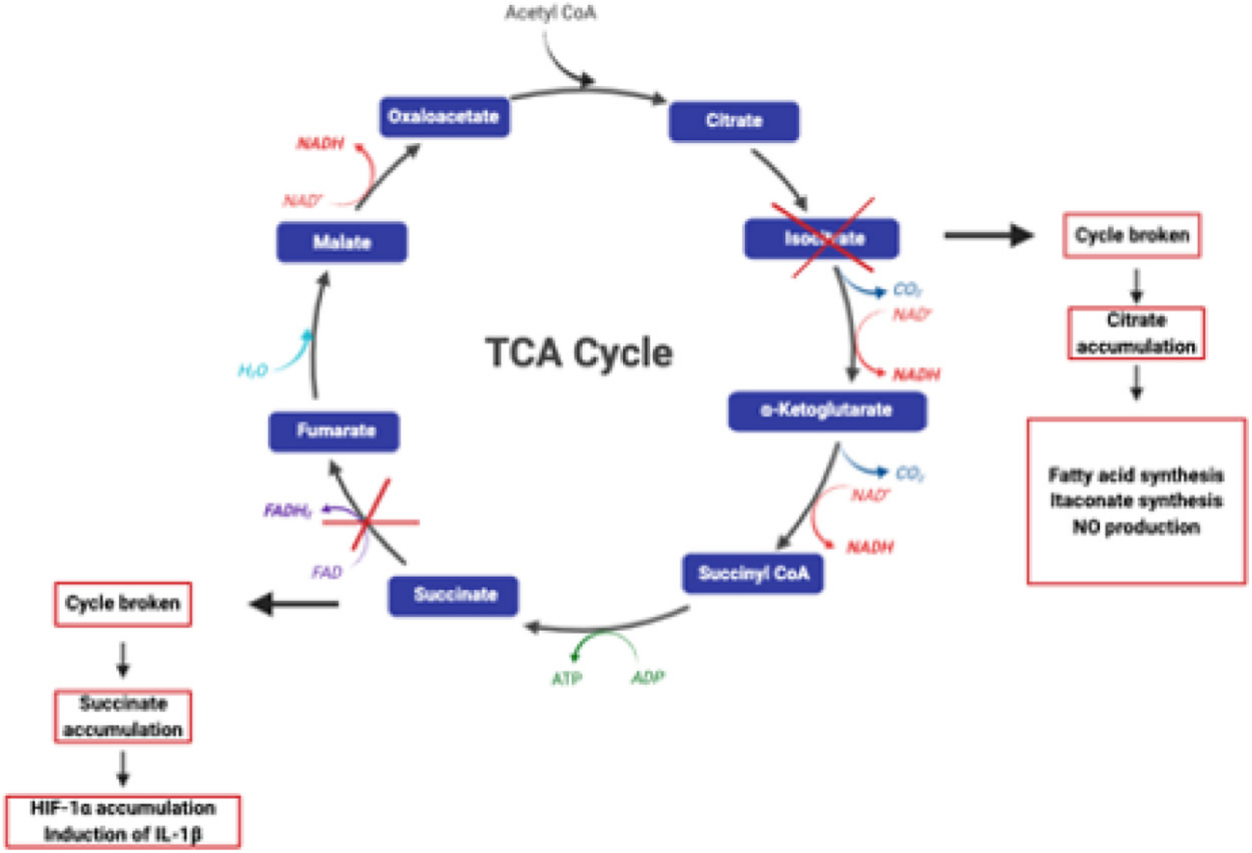
Disruption of the TCA cycle in M1 MØ. In M1-like MØ the TCA cycle is disrupted in two places — after citrate and after succinate leading to the accumulation of both metabolites. Image adapted from O’Neill et al.^2^ and created with BioRender (www.biorender.com).

Furthermore, pyruvate generated by glycolysis fuels the TCA cycle which has been shown to be disrupted at the steps after citrate and succinate generation in pro-inflammatory M1 MØ leading to their subsequent accumulation, in pro-inflammatory M1 MØ (Figure 1).^3^ The resulting citrate can be used for the synthesis of fatty acids, fatty acid derivates such as prostaglandins, the production of nitric oxide (NO)^10^ and the generation of itaconic acid, a metabolite with direct anti-bacterial effects against *M. tuberculosis* (Mtb).^11,12^ The vast majority of studies investigating immunometabolism have used LPS as a stimulatory ligand to induce a pro-inflammatory type of macrophages (for example see ^13–19^). The resulting accumulating succinate stabilises hypoxia-inducible factor 1 α (HIF-1α), resulting in the maintenance of IL-1β production and thus supporting the generation of a pro-inflammatory response by MØ.^17^ HIF-1α is induced by hypoxia and inflammatory stimuli, and triggers and sustains glycolytic and pro-inflammatory pathways.^1,3^ Indeed, HIF-1α activity is essential for the IFN-γ dependent Mtb control.^20^ Lack of HIF-1α resulted in a strongly reduced pro-inflammatory cytokine and NO response and increased susceptibility to Mtb infection in murine *in vitro* and *in vivo* models.^20^

However, the role of MØ cell metabolism during tuberculosis (TB) infection has found great interest recently,^21,22^ recognising a major influence of MØ bioenergetics in the response to mycobacterial pathogens. This driven by recent our increased understanding of the metabolic interplay between immune cells and Mycobacteria that have provided new insights into how their interactions, and ultimately influence disease outcomes and antibiotic-treatment efficacy. Recent studies have shown that, similar to LPS-induced M1 MØ polarisation, Mtb induces a metabolic shift in murine MØ towards aerobic glycolysis.^3,20,23–25^ This shift has been shown to be TLR2-dependent^24^ and furthermore HIF-1α coordinated in IFN-γ activated MØ,^20^ resulting in increased pro-inflammatory MØ effector function. However, this paradigm has been challenged lately as Cumming et al.^26^ reported a downregulation of both, OXPHOS and glycolysis, upon live Mtb infection in human MØ, suggesting the induction of a quiescent energy phenotype by live Mtb in primary cells. Shi et al.^25^ also recently reported a biphasic dynamic of MØ metabolism with an early phase, characterised by M1 MØ polarisation, but a late adaptation post 24 h with transition from glycolysis to OXPHOS, indicating a subsequent downregulation of MØ pro-inflammatory and anti-bactericidal responses. Thus, MØ immunometabolism is clearly an emerging field and further studies elucidating the complexity of metabolic changes induced by pathogens in the host are indicated to improve our understanding. Particularly, in the context of a pathogen of global significance such as Mtb.

Most investigations into cellular immunometabolism to mycobacterial infection have been conducted in the murine model or by using cell lines. However, we believe that such investigations need to be performed in host-matched species for the pathogen being studied, especially one with such exquisite tropism as Mtb/M.bovis. This is further highlighted by the fact that distinct differences in the metabolic profile of murine and human MØ have been identified,^19^ suggesting species-specific differences. So far, studies examining the metabolic potential of bovine MØ are lacking. Furthermore, given the recently described differences in human and bovine pattern recognition receptors (PRR) interaction with defined ligands^27–31^ as well as the differences in MØ-mycobacteria interaction,^32^ we investigated in the current study the basic respiratory parameters and bioenergetics of bovine and human MØ in response to a variety of ligands to shed further light on these differences.

## Materials and methods

### Cell culture

#### Isolation of bovine peripheral blood mononuclear cells (PBMCs)

Blood for peripheral blood mononuclear cells (PBMC) isolation and subsequent MØ generation was collected by puncture of the jugular vein from clinically healthy pure-breed pedigree Holstein Friesian (HF) cows housed at the RVC Bolton Park Farm (Hertfordshire, UK) and Cancourt Farm (Wiltshire, UK). All procedures were carried out under the Home Office license (PPL7009059) approved by the RVC's Ethics and Welfare Committee (URN 2019 1916-3). For biological assays, blood was drawn into sterile glass vacuum bottles containing 10% acid citrate dextrose (ACD) as anticoagulant and isolated as previously described.^28,33^ Serum was collected using vacutainers from the same animals.

#### Maturation and culture of bovine *ex-vivo* derived macrophages

PBMCs were isolated as previously described.^28,33^ To derive MØ, thawed PBMCs were set up in 10 × 10 cm dishes in MØ cell culture media, supplemented with 10% L929 fibroblast cell line supernatant as source of M-CSF (produced by the Werling group, RVC) at 1 × 10^6^ cells ml^−1^ in a total volume of 20 ml and incubated at 37°C with 5% CO_2_. Media was replaced after three days and cells were harvested after 6 days. Cells were scraped of the dishes using cell scrapers (Greiner, UK) and cold PBS. After assessing cell viability by Trypan Blue (Sigma, UK) exclusion, cells were seeded at 1 × 10^6^ ml^−1^ in 96-well plates for further assays.

#### Isolation, maturation, and culture of human PBMCs

Human blood was collected from *V. cephalica* of healthy donors into 50 ml EDTA tubes at LSHTM under a CREB with ethics approval (No 2019 1916-3). Sex and *M. bovis* BCG vaccination status of the donors was recorded. Human PBMCs were subsequently isolated following the protocol as described above for the bovine PBMCs to reduce technical impact. To derive MØ from frozen PBMC stocks, cells were treated as described for the bovine MØ.

### Extracellular flux assay

To accurately investigate the bioenergetic function of bovine and human MØ with an extracellular flux analyser (Seahorse Bioscience, Inc, USA), both cell types were characterised according to the manufacturer's recommended basal and test conditions.^34^ Initial experiments were conducted with an 8-well Seahorse XFp extracellular flux analyser (Agilent, USA) to determine cell seeding density and FCCP concentration (see Supplementary Data). All following experiments investigating metabolic parameters of both cell types upon ligand stimulation, were conducted with a 96-well Seahorse XFe extracellular flux analyser (Agilent, USA).

### Investigation of metabolic parameters

Key metabolic parameters of human and bovine MØ were determined in real time by measuring oxygen consumption rate (OCR) and Extracellular Acidification Rate (ECAR) as a surrogate marker for glycolysis using a Seahorse XFe 96-well extracellular flux analyzer (Agilent, USA). Briefly, *ex-vivo* derived bovine and human MØ were matured as described above and seeded at a density of 1.5 × 10^5^ cells in 180 µl in XFe cell culture microplates (Agilent, USA). Cells were stimulated with LPS (1 ng ml^−1^; Invivogen, USA), FSL-1 (100 ng ml^−1^; Invivogen, USA), recombinant bovine (rbo) TGFβ1 (10 ng ml^−1^, National Institute of Biological Standards and Control (NIBSC), UK) at, PPD *M. bovis* (1 µg ml^−1^, NIBSC, UK) or *M. bovis* BCG Pasteur at an MOI of 10 for 24 h incubation. Following the incubation, cells were washed, and MØ cell culture media was replaced with FCS-and bicarbonate-free DMEM medium supplemented with 4.5 mg ml^−1^ D-glucose and 2 mM glutamine (Agilent, USA) for another 60 min incubation at 37°C without CO_2_. The XFe96 sensor cartridge was hydrated overnight prior to the assay and used to calibrate the analyser. Compounds of the Mito Stress Test kit (Seahorse Bioscience, Inc, USA) target components of the electron transport chain (ETC) were prepared according to the manufacturers’ instructions. After calibration, the cell culture plate was loaded and basal OCR and ECAR were recorded following by sequential addition of the compounds of the Cell Mito Stress Test kit.^34^ Firstly, oligomycin (inhibitor of ATP synthase) was added to reach a final concentration of 1 μM, followed by FCCP (uncoupling agent) at a final concentration of 2.0 μM and subsequently rotenone/antimycin A (inhibitors of complex I and complex III of the respiratory chain, respectively) to reach a final concentration of 0.5 μM per wells (See Supplementary Figure 1). Data was recorded in wave controller software (V2.4) and exported to Excel (V16.16.14) and GraphPad Prism (Dotmatics, V8.4.3). Parameters are then extrapolated using the multi-report generator files from Agilent Technologies, which automatically calculate proton leak and spare respiratory capacity using measured parameters during the assay, such as ATP production, maximal respiration, and non-mitochondrial respiration.

### Measurement of nitric oxide

Measurement of NO concentration from cell supernatants from bovine and human macrophage experiments was carried out using Griess reagents (Promega, UK) according to the manufacturer's protocol. Briefly, a three-fold standard curve of 128uM sodium nitrite in 10% FCS MQ media and sample supernatants in duplicates were placed in a flat 96-well plate (Greiner, UK) and mixed with equal volumes of solution A and B of Griess reagents. Following 10 min of incubation, absorbance was measured at 550 nm using a spectrophotometer (Spectramax M2, Molecular Devices, UK).

### Statistical analysis

Statistical analysis and graphs were generated using GraphPad Prism software package. All results were checked for normal distribution and equal variance assumption and are presented as mean +/- standard deviation. Datasets that passed normality tests and equal variance assumptions, but appeared to have outliers, were log transformed and re-analysed to verify no change in statistical results. Datasets that were additionally log transformed to account for outliers are clearly marked within the manuscript. Data were analysed using a two-way ANOVA with Tukey's HSD post-hoc comparison. Statistical significance was defined as *p* < 0.05(*), *p* < 0.01(**), *p* < 0.001(***) and *p* < 0.0001(****).

## Results

### 2-Deoxy-D-glucose Inhibits glycolysis in both, bovine and human MØ

Using the hexokinase inhibitor 2-Deoxy-D-glucose (2-DG) to inhibit the first step of glycolysis has been shown to be a useful tool to examine glycolytic parameters more closely.^7^ Indeed, it is a well-known fact that 2-DG inhibits glycolysis, and it can therefore be used as a functional control. Here, we first examined the ability of 2-DG to control MØ metabolism and to examine whether its effects were similar in both species. MØ of both species were incubated for 24 h with 2-DG before assessing ECAR (as surrogate measure of glycolysis). As reported for other species, 2-DG decreased OCR and strongly decreased glycolysis in MØ of both species to a greater extent than other ligands (LPS and FSL-1), which were used for comparison in the same experiments (See supplementary data).

### Bovine and human MØ exhibit similar mitochondrial bioenergetics pattern upon LPS stimulation

Most studies have assessed MØ cell metabolism where the M1 phenotype was generated upon LPS stimulation. Thus, we examined cellular bioenergetics in bovine and human MØ in response to the TLR4 agonist LPS.

**Figure 2.**
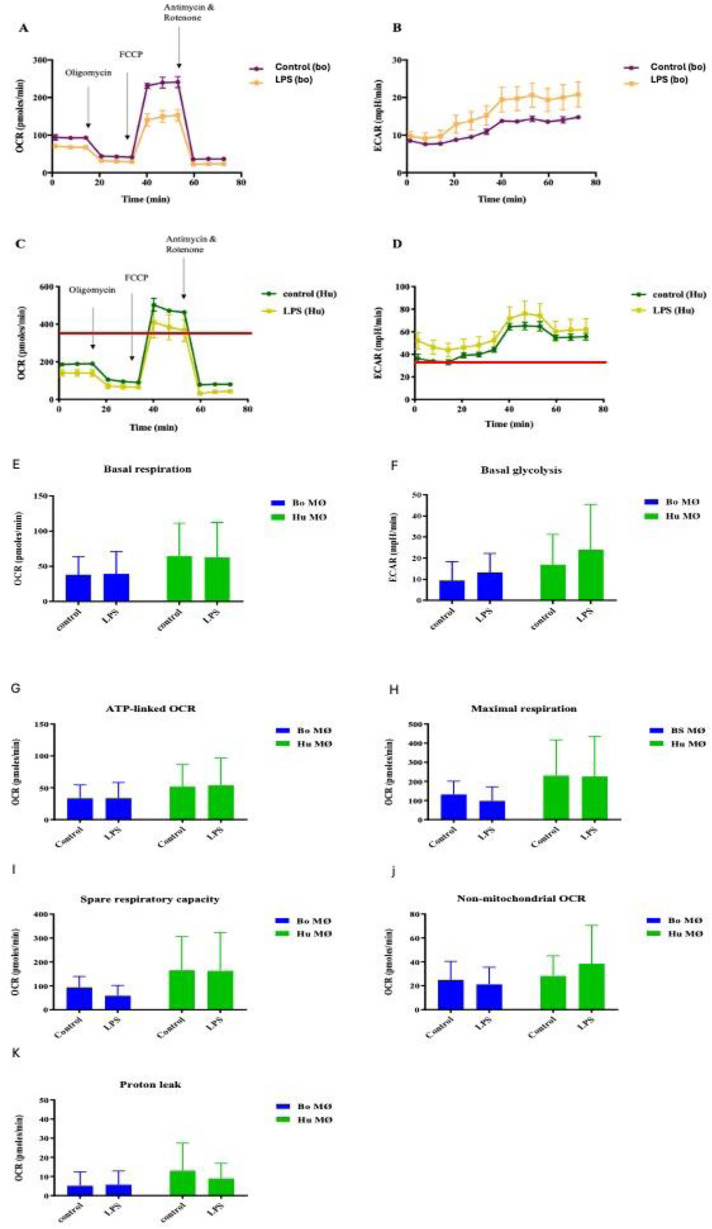
Basal respiration and glycolysis of bovine and human MØ to LPS stimulation. Representative plot of OCR (A and C) and ECAR (B and D) of a bovine MØ (Bo) (A and B) and a human (Hu) MØ sample (C and D) upon stimulation with 1 mM 2-DG (Sigma-Aldrich, UK) for 24 h subjected to a Cell Mito Stress test (Agilent Technologies, USA) and measured with the Seahorse XFe extracellular flux analyser (Agilent Technologies, USA) over 75 min. Following measurement of basal respiration, OCR and ECAR are recorded after injection of 1 μM Oligomycin, followed by 2.0 μM FCCP injection and 0.5 μM of antimycin A and rotenone injection (injection time points are indicated by arrows). Basal respiration and glycolysis of bovine (Bo) and human (Hu) MØ upon stimulation with 1μg ml^−1^ LPS (Invivogen, USA) for 24 h measured using the Seahorse XFe extracellular flux analyser (Agilent Technologies, USA) prior to a Cell Mito stress test (Agilent Technologies, USA). Values are the mean of three independent experiments with three technical repeats each and are shown as +/- SD. Statistical analysis was performed using paired Student's t-test relative to controls in GraphPad Prism V8 (GraphPad Inc., USA). Statistically significant difference is indicated by asterisk (* =*p* < 0.05). Values are presented o *n* = 8 for Bo MØ and *n* = 4 for human MØ.

Upon LPS stimulation, the overall respiratory profile differed between control and stimulated groups for both species, though only very mildly for basal respiration, resulting in a minor decrease of OCR for bovine and human MØ (Figure 2A and 2C), whereas glycolysis was increased for both species (Figure 2B and 2D). While the magnitude of difference varied greatly between the two species, the overall trend remained the same for each species.

Levels of OCR are an indicator of OXPHOS. Initially basal respiration was measured before the injection of cell Mito stress test compounds to determine specific respiratory parameters. Basal respiration was similar in LPS-stimulated MØ and their corresponding controls for both species (Figure 2E). ECAR, a surrogate measure of glycolysis, was slightly elevated in both species upon LPS stimulation (Figure 2F).

For both species, responses to LPS showed minimally impaired respiratory parameters upon LPS stimulation, (Figure 2G, H, I, J and K). The only exemptions could be seen in human MØ, where a tendency towards a minimal increase of non-mitochondrial respiration (Figure 2(j)) and decline of proton leak was observed (Figure 2(k)). In summary, considering the data obtained from all individuals, it appeared that mitochondrial bioenergetics was not significantly altered in either species upon LPS stimulation. However, it is noteworthy that basal parameters were consistently the highest for human MØ. The strong individual variation between samples was reflected in all respiratory parameters, and may have prevented the differences to reach significant value.

### Bovine and human MØ exhibit similar mitochondrial bioenergetics upon FSL-1 stimulation

**Figure 3.**
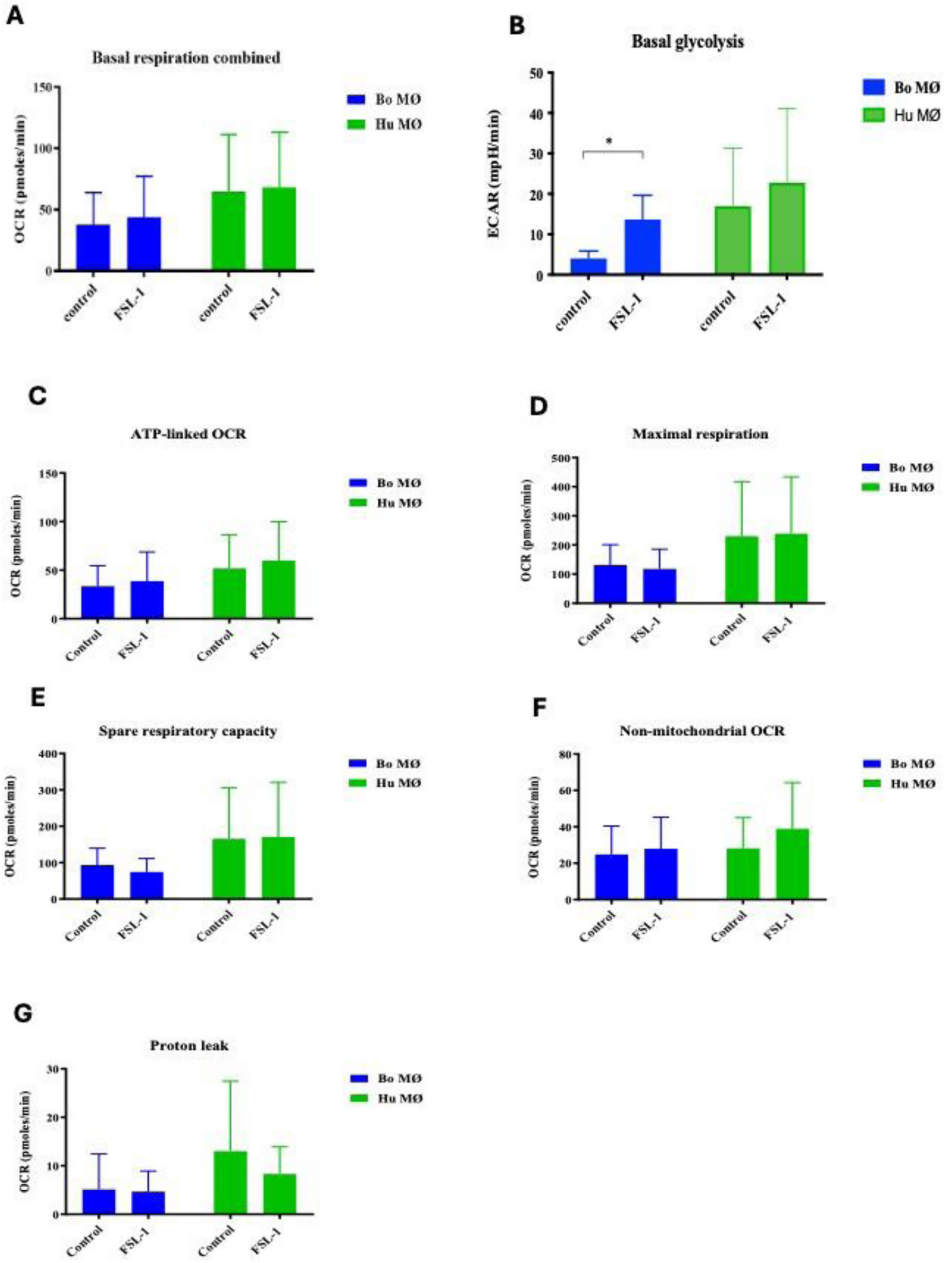
Basal respiration and glycolysis of bovine and human MØ to FSL-1 stimulation. Basal respiration (A) and glycolysis (B) of bovine and human (Hu) MØ (*n* = 4 per species) upon stimulation with 100 ng ml^−1^ FSL-1 (Invivogen, USA) for 24 h measured with the Seahorse XFe extracellular flux analyser (Agilent Technologies, USA) prior to a Cell Mito stress test (Agilent Technologies, USA). C-G show the results of further respiratory parameters analysis. Values are the mean of the independent experiments with three technical repeats each and are shown as +/- SD. Statistical analysis was performed using paired Student's t-test relative to controls in GraphPad Prism V8 (GraphPad Inc., USA). Statistically significant difference is indicated by asterisk (* =*p* < 0.05).

Having observed a similar mitochondrial bioenergetic profile upon LPS stimulation between the two species, we next investigated the response to the synthetic TLR-2 agonist FSL-1. Breed specific responses to FSL-1 has been described by us and others.^27,32^ A similar profile of mitochondrial bioenergetics as observed upon LPS stimulation was detected (data not shown). Basal respiration equalled among controls and stimulated groups for both species (Figure 3A) and glycolysis rates were elevated in the stimulated groups (Figure 3B). This increase was significantly for bovine MØ (Figure 3B). All other respiratory parameters did not show significant differences (see Figure 3C - 3G).

### Enhanced activation of respiratory parameters upon PPD of *M. bovis* stimulation in human MØ

**Figure 4.**
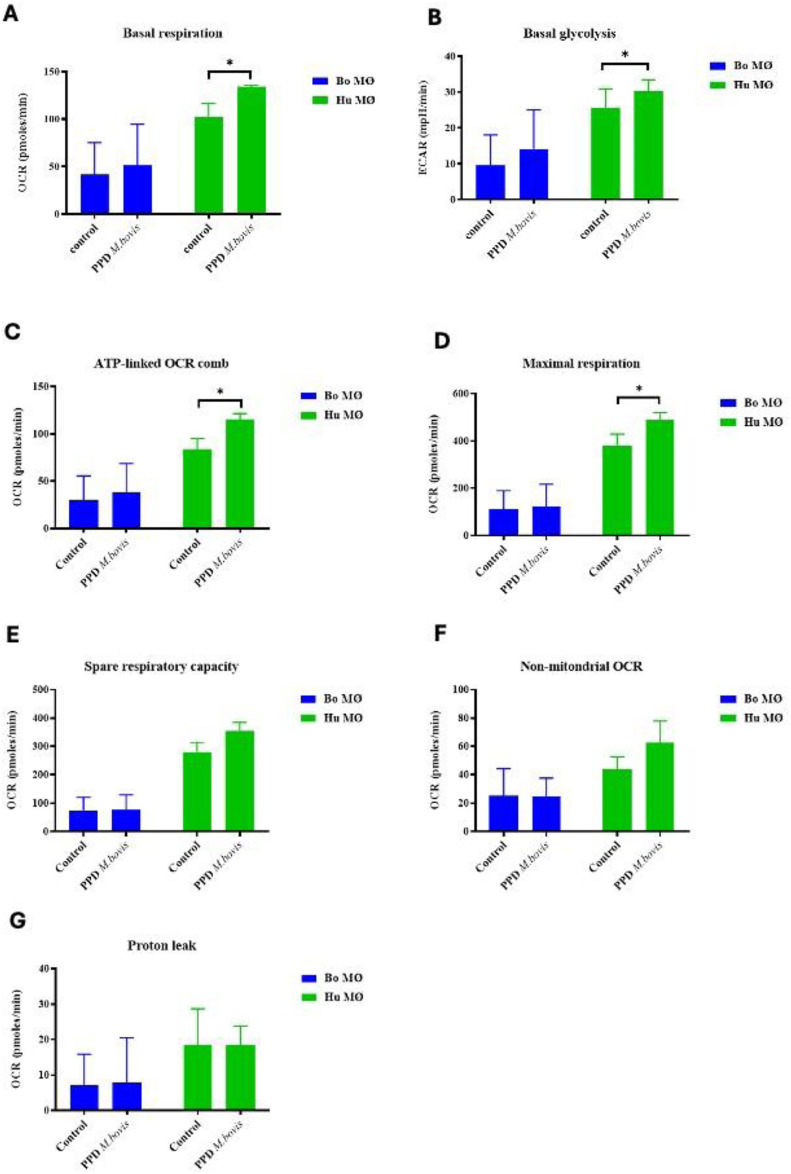
Basal respiration and glycolysis of bovine and human MØ to PPD of *M. bovis* stimulation. Basal respiration (A) and glycolysis (B) of bovine (Bo, *n* = 8) and human (Hu) MØ (*n* = 4) upon stimulation with 1 μg ml^−1^ PPD of *M. bovis* (NIBSC, UK) for 24 h measured with the Seahorse XFe extracellular flux analyser (Agilent Technologies, USA) prior to a Cell Mito Stress test (Agilent Technologies, USA). Remaining parameters are shown in Fig. 4 C-G. Values are of the mean of independent experiments with three technical repeats each and are shown as +/- SD. Statistical analysis was performed using paired Student's t-test relative to controls in GraphPad Prism V8 (GraphPad Inc., USA). Statistically significant differences are indicated by asterisk (*=*p* < 0.05).

As experiments with the extracellular flux analyser were only possible under Biosafety level 2 laboratory conditions, purified protein derivative (PPD) derived from *M. bovis* (NIBSC, UK) was used as a substitute rather than using live *M. bovis*. Interestingly, whereas basal respiration seemed to be only minimally increased in bovine MØ, it was significantly increased in human MØ (Figure 4A). Similarly, basal glycolysis was not significantly elevated for bovine MØ, but for human MØ only (Figure 4B), indicating a species difference upon stimulation with PPD of *M. bovis*. Furthermore, with the exemption of proton leak, all respiratory parameters were elevated in human MØ, with significant differences for ATP-linked OCR and maximal respiration (Figure 4C and D), suggesting a strong OXPHOS activation upon PPD of *M. bovis* stimulation in human MØ, potentially further contributing to the differences seen recently how MØ from both species handle Mycobacteria.^30,32^ The remaining parameters (shown in Figure 4E – G) did not show any significant differences.

### Bovine and human MØ exhibit similar mitochondrial bioenergetics upon PPD of M. *tuberculosis* stimulation

**Figure 5.**
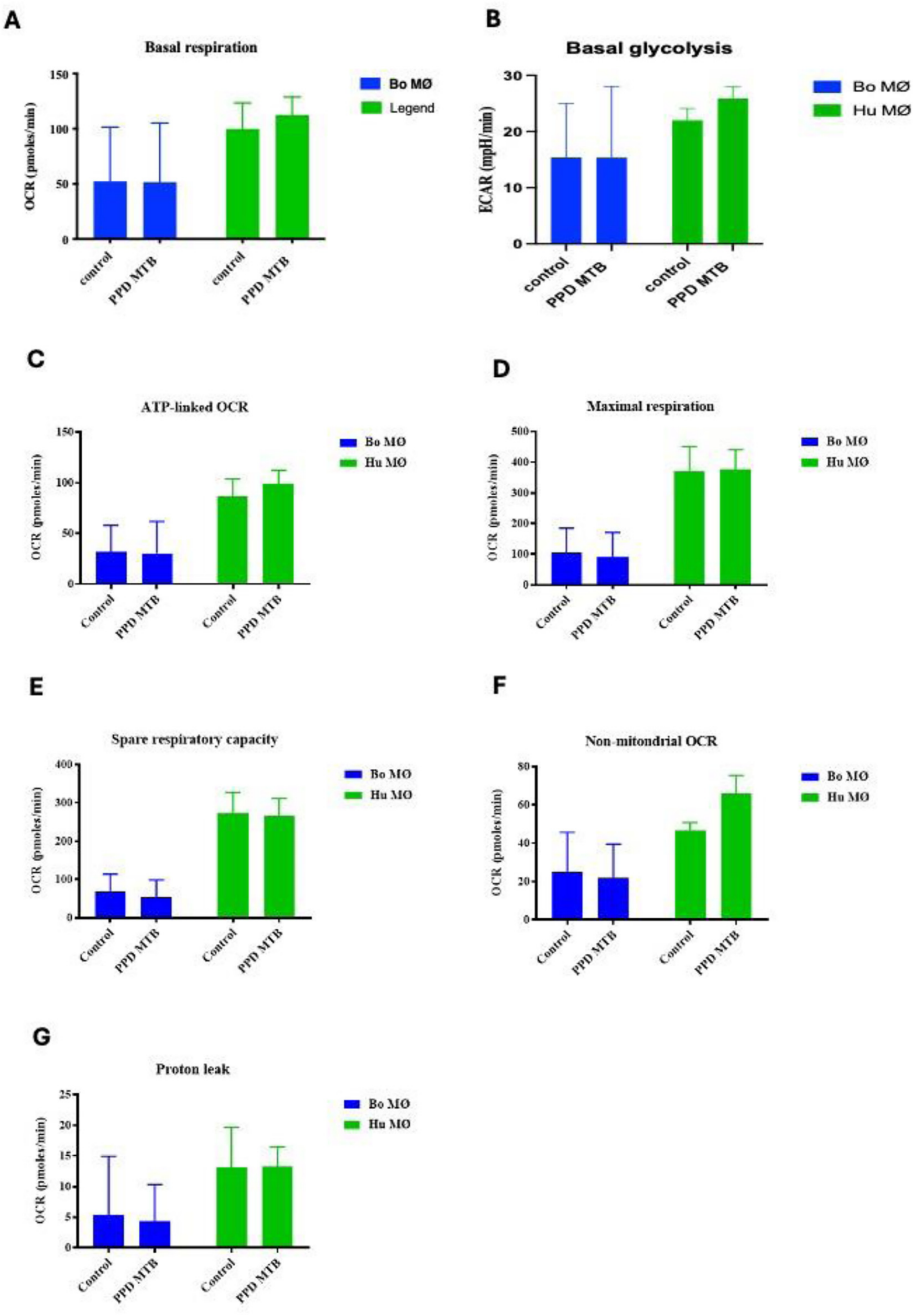
Basal respiration and glycolysis of bovine and human MØ to PPD of Mtb stimulation. Basal respiration (A) and glycolysis (B) of bovine (Bo, *n* = 6) and human (Hu) MØ (*n* = 3) upon stimulation with 1 μg ml^−1^ PPD of Mtb (NIBSC, UK) for 24 h measured with the Seahorse XFe extracellular flux analyser (Agilent Technologies, USA) prior to a Cell Mito Stress test (Agilent Technologies, USA). Remaining parameters are shown in Fig. 4 C-G Values are the mean of independent experiments with three technical repeats each and are shown as +/- SD. Statistical analysis was performed using paired Student's t-test relative to controls in GraphPad Prism V8 (GraphPad Inc., USA). Statistically significant differences are indicated by asterisk (*=*p* < 0.05).

To assess mitochondrial bioenergetics in response to *M. tuberculosis* (Mtb)*,* PPD derived from Mtb (NIBSC, UK) was also used as comparable substitute for live virulent Mtb. In general, human MØ showed higher values for all parameters tested compared to bovine MØ (Figure 5A – G). As observed before for PPD of *M. bovis*, human MØ showed a tendency for a higher basal respiration and glycolysis upon PPD of Mtb stimulation (*p* = 0.06; Figure 5A and 5B), however compared to PPD derived from *M. bovis,* this did not reach levels of significants for any parameter tested (Figure 5A - G). Indeed, all respiratory parameters analysed for bovine and human MØ showed similar levels under control or stimulated condition, with the only exemption being an non-significant increase in non-mitochondrial respiration in human MØ (Figure 5F; *p* = 0.06).

### Effect of metabolic changes on nitric oxide production

**Figure 6.**
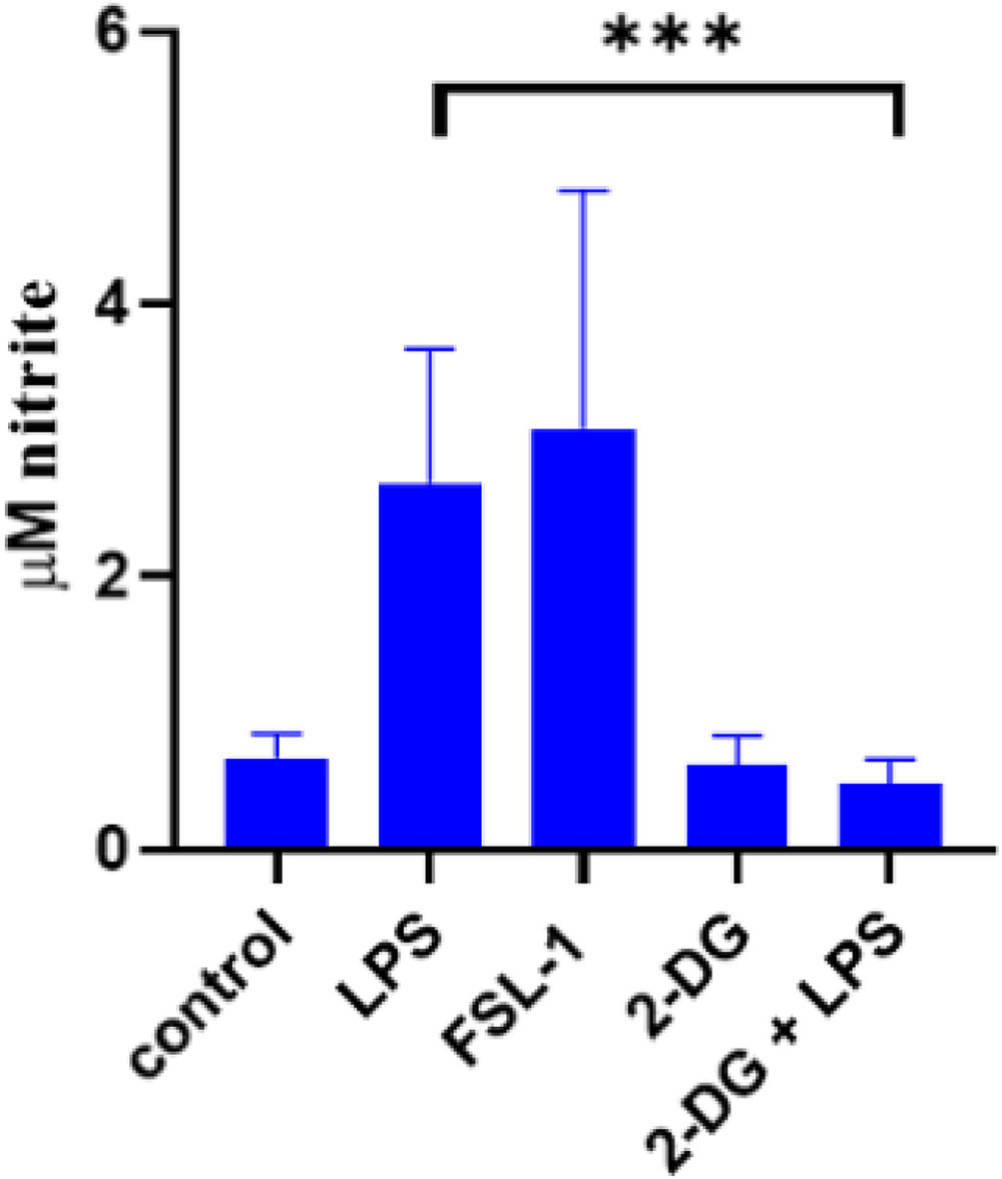
No production by bovine MØ in response to various ligands prior to extracellular flux assay analysis. Bovine MQ generated were stimulated with 1 mg ml^−1^ LPS, 100 ng ml^−1^ FSL-1 and 1 mM 2DG for 24 h. Thereafter, supernatants were collected before cells were subjected to Mito stress test, and NO production by Griess assay (Promega, UK). Statistical analysis was performed using a repeated measures two-way ANOVA and Sidak's post hoc comparison using GraphPad Prism software package (Version 8, GraphPad Inc., USA). All samples were run in duplicates and mean +/- SD are shown. (*=*p* < 0.05, **=*p* < 0.01, ***p < 0.001, ****=*p* < 0.0001).

Previously, we demonstrated clear differences in the production of reactive oxygen and reactive nitrogen between human and bovine MØ to PRR ligands,^35^ and this was expanded more recently by us and others also in response to either *M. bovis* and Mtb (^30,32,36,37^). Given that NO was recently describe as modulator of metabolic programming, and impacts on glycolysis, we assessed the outcome of cellular incubation to 2-DG (which decreases the inflammatory response^7^) as well as LPS and FSL-1 in supernatants of wells before MØ underwent Cell Mito Stress test assessment. Both, LPS and FSL-1 increased production of NO significantly. As expected, NO production was reduced to base levels upon addition of 2-DG to LPS stimulation on bovine MØ (Figure 6).

## Discussion

The aim of this study was to explore some basic immune-metabolic functionality in bovine and human MØ in response to a variety of ligands. Most studies in this field have been conducted using murine MØ or cell lines to investigate metabolic changes, however functional differences between MØ types and species have been reported.^19,38^ No prior investigations into the cellular bioenergetics of bovine MØ have been made so far. However, given the generally reduced cytokine and NF-kB response to TLR ligands seen in bovine MØ,^29,31,39^ we wanted to assess whether a similar phenomenon could be observed comparing the metabolism of bovine MØ and human MØ using a Seahorse extracellular flux analyser and a Cell Mito Stress test (both Agilent Technologies, USA) which allowed the determination of key parameters of mitochondrial respiration in both species.

Overall, there was a trend for increased glycolysis in response to all ligands. An increase in glycolysis upon stimulation with LPS, but also to mycobacteria is considered a hallmark event in activated MØ.^1,4^ This increase in glycolytic pathways allows for rapid energy production and triggers host defence pathways such as pro-inflammatory cytokine and effector molecule expression.^2–4^ For instance, metabolic reprogramming has been shown to be essential in control of mycobacterial infection.^20,23–25^ Inhibition of glycolysis by 2-DG, which was used in a control experiment here (see Supplmentary figures), has been shown to enhance mycobacterial growth, suggesting that glycolysis is required for limiting Mtb growth.^40^ Here NO production, an essential antimicrobial, was also found to be ameliorated upon 2-DG treatment in all cell types analysed indicating that depleting glycolysis would have similar effects for mycobacterial control in human and bovine MQ.

### Species-specific differences in the metabolic potential and respiratory profile of human and bovine macrophages

The overall response pattern did not vary significantly between the species. Highest basal values were measured in human MØ compared to bovine MØ. Higher basal values of human MØ were also found in the study by Vijayan et al. in comparison to murine bone marrow-derived MØ.^19^ However, this was likely just a reflection of the MØ type used under specific cell culture conditions, whereas the focus of the present study was to examine the response of primary bovine and human MØ generated in the same manner to identical ligands. Indeed, the source of MØ generation, their method of culture and maturation, differential stimulation periods and dose of infections have been shown to strongly impact the metabolic profile of MØ and subsequently the outcome of the response. In our present study a consistent approach was used, as MØ of both species were generated in the same manner and always stimulated for 24 h. Nonetheless, through this approach some time-dependent metabolic changes as reported by Shi *et al*. may have been missed.^25^ Additionally, the MØ lineage of the same species seemed to impact on their metabolic profile. Huang et al. found differences in the metabolism of interstitial and alveolar MØ during Mtb infection, with the former showing a higher glycolytic activity and the latter skewed to fatty acid oxidation.^40^

### The differences in respiratory profiles of bovine and human macrophages in response to different innate immune stimuli

When comparing respiratory parameters between the bovine and human species, no distinct differences were detected. This differed from results published in a recent study, reporting significant differences in the metabolic profile of human and murine MØ upon LPS stimulation, showing impaired mitochondrial bioenergetics in the murine samples.^19^ There is growing evidence linking MØ metabolism to the production of inflammatory mediators.^7^ Upon stimulation with various ligands, MØ undergo metabolic reprogramming, resulting in impaired OXPHOS and increased glycolysis and the latter has been found essential for pro-inflammatory MØ function.^2,4,7^ Here, we observed similar mitochondrial bioenergetics in MØ generated from both species in response to LPS, FSL-1, and PPD of Mtb. Similar to others, we observed a rapid increase in glycolysis, as measured by the surrogate ECAR, to all the TLR agonists used.^41^ However, only a mild inhibition of mitochondrial respiration in response to agonists was found in this study. Interestingly, OXPHOS, linking the TCA to ATP production, was found to be non-essential for MØ to sustain inflammatory polarisation, contrary to glycolysis whose upregulation is needed for inflammatory function and cell survival.^42^ Recently, NO has been demonstrated to be a central modulator of this metabolic switch. The commitment of MØ to glycolysis after stimulation could be a direct effect of the TLR-ligand interaction, leading to expression of inducible nitric oxide synthase and subsequently NO production. NO can act as inhibitor of Complex I and reversibly, Complex IV of the ETC.^18,42^ Increased levels of NO production have been observed for mycobacterial infection as well as in the present study in supernatants from cells subsequently used for metabolism assays (Figure 6).^30^ As NO inhibits mitochondrial electron transport, therefore blocking oxygen consumption and coupled ATP production,^43^ this may explain the decreased mitochondrial respiration upon agonist stimulation observed in some samples. While this effect may represent one of the innate defence mechanisms MØ can apply to deal with mycobacterial infection, it can also lead to a rapid use of energy reserves within the cells.

### The metabolic changes in macrophages in response to different mycobacterial PPD

Interestingly, several authors have described different responses of either human and bovine MØ exposed to either mycobacterial species^32^ as well differences how bacterial infection is dealt with in cattle.^37,44^ Very simplified, it seems that bovine MØ can deal with Mtb as pathogen, but not *M. bovis*, whereas human MØ are infected by both mycobacterial species.^32,45^ These observations triggered our experiments to assess whether observed changes might be at least in part explained by metabolic changes in MØ from either species. According to our data, a stronger activation of mitochondrial respiration was observed to PPD of *M. bovis* in human MØ. Cummings et al. also observed an increase in the overall OCR measured in the Cell Mito Stress test and some respiratory parameters such as maximal respiration and spare respiratory capacity upon *M. bovis* BCG stimulation of human MØ, in contrast to exposure to live and dead Mtb.^26^ It could be hypothesised that *M. bovis* activates human MØ more strongly, which is further supported by the absence of a significantly stronger activation upon PPD of Mtb stimulation in human MØ. In their study, Mtb drastically decreased MØ respiratory parameters which was found to be MOI dependent.^26^ However, it has to be kept in mind that Cummings et al. used live bacteria,^26^ in contrast to the present study using PPD derivative only. Indeed, the authors observed distinct differences in the respiratory capacity of MØ to live and dead Mtb and *M. bovis* BCG, and additionally these were dependent on the MØ type (cell line vs primary cells) and dose of infection.^26^

### General conclusions and limitation

The results presented here were exploratory, elucidating a basic respiratory profile of bovine MØ in comparison to human MØ. Further investigations into specific metabolic pathways using live bacteria are indicated to allow for further assumptions, but this was clearly the strongest limitation of the project. We were unable to directly perform he experiment using live bacteria due to the lack of a device in a Cat 3 environment and considered the ability to perform these assays using inactivated bacteria as artificial. Thus, using defined ligands seemed to be the most appropriate way forward to obtain a basic understanding of the metabolic functionality of MØ of both species. A further limitation was that we analysed ECAR as substitute for glycolysis. In further experiments, the GlycoPER test needs to be used to assess glycolysis directly. However, considering these preliminary results, it can be hypothesised that bovine MØ have similar bioenergetic profile to human MØ upon stimulation with the most commonly used PAMP, such as LPS and others used herein, and thus might serve as a useful comparative tool to study MØ metabolism further.

## Supplemental Material

sj-pptx-1-ini-10.1177_17534259241296630 - Supplemental material for Respiratory bioenergetics is enhanced in human, but not bovine macrophages after exposure to *M. bovis* PPD: Exploratory insights into overall similar Cellular Metabolic Profiles Supplemental material, sj-pptx-1-ini-10.1177_17534259241296630 for Respiratory bioenergetics is enhanced in human, but not bovine macrophages after exposure to *M. bovis* PPD: Exploratory insights into overall similar Cellular Metabolic Profiles by Marie-Christine Bartens, Sam Willcocks, Dirk Werling and Amanda J Gibson in Innate Immunity

sj-docx-2-ini-10.1177_17534259241296630 - Supplemental material for Respiratory bioenergetics is enhanced in human, but not bovine macrophages after exposure to *M. bovis* PPD: Exploratory insights into overall similar Cellular Metabolic Profiles Supplemental material, sj-docx-2-ini-10.1177_17534259241296630 for Respiratory bioenergetics is enhanced in human, but not bovine macrophages after exposure to *M. bovis* PPD: Exploratory insights into overall similar Cellular Metabolic Profiles by Marie-Christine Bartens, Sam Willcocks, Dirk Werling and Amanda J Gibson in Innate Immunity

## Abbreviations

2-DG: 2-Deoxy-D-Glucose
ACD: Acid Citrate Dextrose
ANOVA: Analysis of Variance
ATP: Adenosine Tri-phosphate
BCG: *M. bovis* strain Bacillus-Calmette Guérin
BS: Brown Swiss
DMEM: Dulbecco's Minimal Essential Medium
ECAR: Extracellular Acidification Rate
EDTA: Ethylenediaminetetraacetic Acid
ETC: Electron Transport Chain
FCCP: Carbonyl Cyanide-p-(trifluoromethoxy) phenylhydrazone
FSL-1: Fibroblast–stimulating lipopeptide-1, TLR-2/6 ligand representing N-terminus of LP44 from *Mycoplasma salivarium*
HF: Holstein Friesian
HIF-1α: Hypoxia Inducible Factor-1α
HSD: Honestly Significant Difference (for Tukey's HSD test)
IFN-γ: Interferon-γ
IL-4: Interleukin-4
LPS: Lipopolysaccharide
LSHTM: London School of Hygiene and Tropical Medicine
M1: Type 1 Macrophages
M2: Type 2 Macrophages
MM: Macrophage Media
MØ: Macrophage
MOI: Multiplicity of Infection
Mtb: *M. tuberculosis*
mTOR: Mammalian Target of Rapamycin
NADPH: Reduced Nicotinamide Adenine Dinucleotide Phosphate
NIBSC: National Institute of Biological Standards and Control
NO: Nitric Oxide
OCR: Oxygen Consumption Rate
OXPHOS: Oxidative Phosphorylation
PBMC: Peripheral Blood Mononuclear Cells
PBS: Phosphate Buffered Saline
PPD: Purified Protein Derivative
PPP: Pentose Phosphate Pathway
PRR: Pattern Recognition Pathway
ROS: Reactive Oxygen Species
TB: Tuberculosis
TCA: Tricarboxylic Acid Cycle
TGF-β1: Transforming Growth Factor - β1
TH1: T helper 1 cells
TH2: T helper 2 cells
TLR: Toll-like Receptor
