# Supplementary material for: Respiratory bioenergetics is enhanced in human, but not bovine macrophages after exposure to M. bovis PPD: Exploratory insights into overall similar Cellular Metabolic Profiles
Source: Innate Immun. 2024 Nov 20;30(6-8):136–49. doi: 10.1177/17534259241296630 (PMC11577332; doi:10.1177/17534259241296630)
Supplement: sj-pptx-1-ini-10.1177_17534259241296630 - Supplemental material for Respiratory bioenergetics is enhanced in human, but not bovine macrophages after exposure to M. bovis PPD: Exploratory insights into overall similar Cellular Metabolic Profiles [file sj-pptx-1-ini-10.1177_17534259241296630.pptx]

## Slide 1
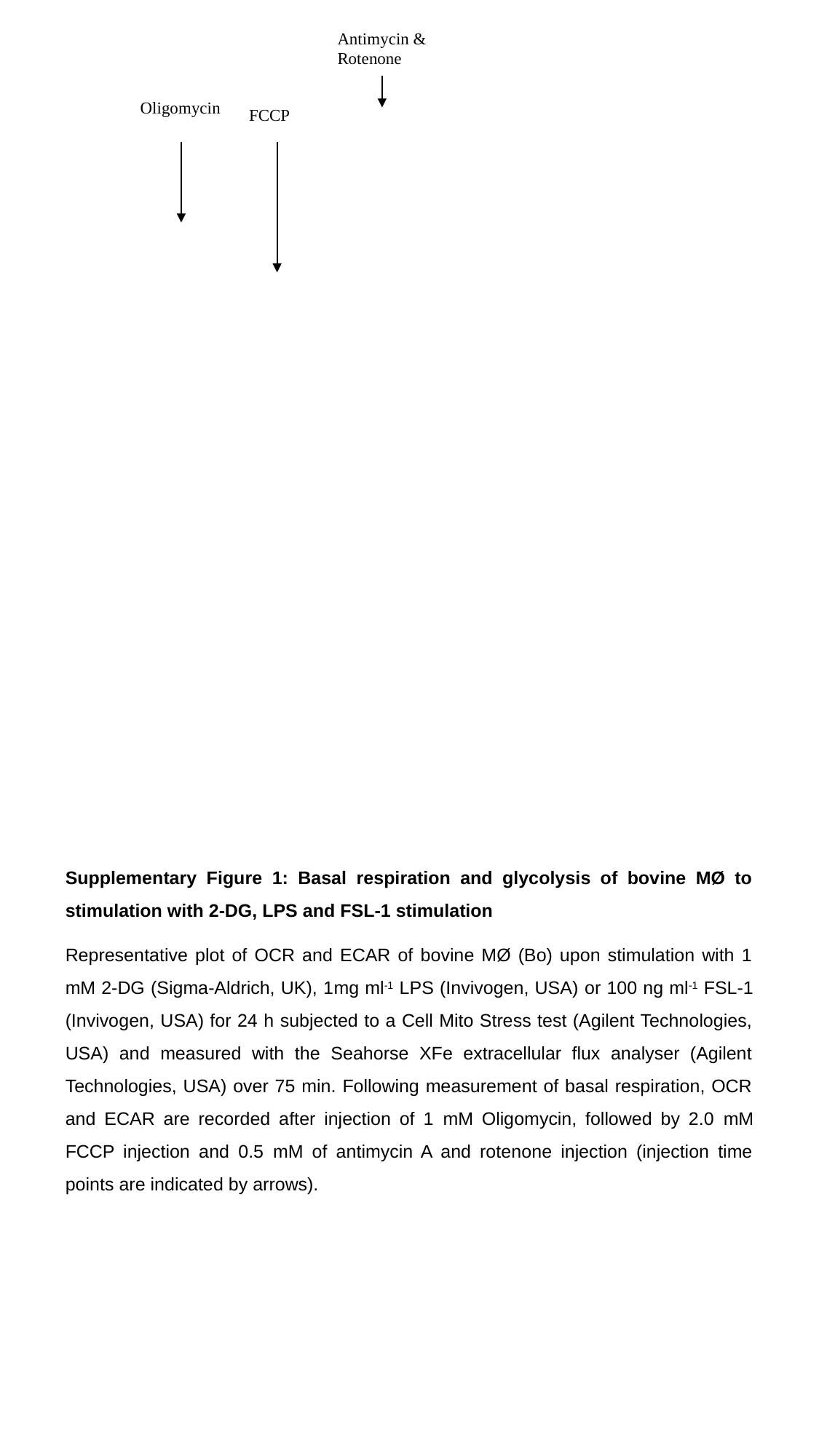

Antimycin & Rotenone
Oligomycin
FCCP
Supplementary Figure 1: Basal respiration and glycolysis of bovine MØ to stimulation with 2-DG, LPS and FSL-1 stimulation
Representative plot of OCR and ECAR of bovine MØ (Bo) upon stimulation with 1 mM 2-DG (Sigma-Aldrich, UK), 1mg ml-1 LPS (Invivogen, USA) or 100 ng ml-1 FSL-1 (Invivogen, USA) for 24 h subjected to a Cell Mito Stress test (Agilent Technologies, USA) and measured with the Seahorse XFe extracellular flux analyser (Agilent Technologies, USA) over 75 min. Following measurement of basal respiration, OCR and ECAR are recorded after injection of 1 mM Oligomycin, followed by 2.0 mM FCCP injection and 0.5 mM of antimycin A and rotenone injection (injection time points are indicated by arrows).
